# Metformin-Enhanced Secretome from Periodontal Ligament Stem Cells Promotes Functional Recovery in an Inflamed Periodontal Model: In Vitro Study

**DOI:** 10.3390/jfb16050177

**Published:** 2025-05-13

**Authors:** Han Na Suh, Ju Young Ji, Jung Sun Heo

**Affiliations:** 1 Korea Institute of Toxicology, 30 Baekhak1-gil, Jeongeup 56212, Jeollabuk-do, Republic of Korea; 2 Department of Maxillofacial Biomedical Engineering, College of Dentistry, Kyung Hee University, 26 Kyunghee-daero, Dongdaemun-gu, Seoul 02447, Republic of Korea

**Keywords:** metformin, periodontal ligament stem cells, preconditioning, secretome, periodontal regeneration

## Abstract

Objective: Secretory factors, termed the secretome, in the conditioned medium (CM) from dental mesenchymal stem cells (MSCs) have shown anti-inflammatory, anti-apoptotic, and tissue regenerative potential. This cell-free product could be further developed by preconditioning cells with various biochemical agents, which lead to a change in secretome and CM profiles. Among the favorable candidates for CM production, metformin as an anti-diabetic medication is currently considered a potential agent for dental hard tissue and periodontal regeneration. Here, we aimed to assess the composition of CM from periodontal ligament stem cells (PDLSCs) grown in metformin-preconditioned media (Met-CM) compared to normal PDLSC-CM and assess the ability of Met-CM to recover the function of inflamed PDLSCs. Methods: Met-CM and normal CM were collected from PDLSCs grown with or without 50 µM metformin, respectively, under healthy culture conditions. Mass spectrometry and liquid chromatography–tandem mass spectrometry (LC–MS/MS) were performed to comparatively evaluate the proteomic profiles in PDLSC-CM versus Met-CM. We then treated the PDLSC cultures with lipopolysaccharide (LPS) from *Porphyromonas gingivalis* to induce inflammation and evaluated the osteogenic/cementogenic differentiation in the presence of Met-CM or normal PDLSC-CM by assessing alkaline phosphatase activity, intracellular calcium levels, and mRNA expression of osteogenic and cementogenic factors, including RUNX2, OCN, OSX, and CEMP-1. Subsequently, we performed RNA sequencing to identify transcriptomic changes in the treated cells. Results: We identified 202 differentially expressed proteins, 175 of which were significant, in Met-CM versus normal PDLSC-CM. Among the analyzed groups, the top three protein classes were protein-binding activity modulator, cytoskeletal protein, and extracellular matrix (ECM) protein. Treatment of PDLSCs with LPS significantly attenuated ALP activity, [Ca^2+^]_i_, and the mRNA expression levels of RUNX2, OCN, OSX, and CEMP-1, whereas treatment with Met-CM alone markedly enhanced PDLSC differentiation activity compared with the control. Moreover, osteogenic/cementogenic differentiation of the LPS-treated PDLSCs was recovered through incubation in Met-CM. Transcriptomic analysis identified 511 and 3591 differentially expressed genes in the control versus Met-CM and LPS versus LPS + Met-CM groups, respectively. The enrichment of biological processes includes positive regulation of DNA-templated transcription and skeletal system morphogenesis in the control versus Met-CM comparison, as well as positive regulation of transcription from the RNA polymerase II promoter and negative regulation of the apoptotic process in the LPS versus LPS + Met-CM comparison. Molecular function analysis demonstrated the enrichment of protein-binding terms among the DEGs from each comparison. Conclusions: Metformin preconditioning enhanced the recovery effect of PDLSC-CM on LPS-induced inflamed PDLSCs. These findings suggest that metformin preconditioning could represent a practical formula for PDLSC-secretome, which may contribute to the development of future cell-free periodontal regenerative strategies.

## 1. Introduction

It is widely recognized that dental stem cells such as dental pulp stem cells (DPSCs), periodontal ligament stem cells (PDLSCs), dental follicle cells (DFCs), and stem cells from human pulp of exfoliated deciduous teeth (SHED) and apical papilla (SCAPs) are a key component of regenerative dentistry, with the competence to release growth factors, cytokines, and various signaling molecules that are thought to promote dental tissue regeneration [1,2,3]. These stem cell-derived paracrine factors, referred to as the secretome, in conditioned medium (CM) include tissue-protective and regenerative molecules that can act as cell-free therapeutics [4]. A trend toward the use of stem cell CM in periodontal tissue regeneration research is also emerging, with results suggesting that indirect stem cell therapy shows effects consistent with those of stem cell transplantation [5,6]. Among the possible sources of dental stem cell CM, periodontal ligament stem cells (PDLSCs) are most effective at supplying a large number of secretory molecules for periodontal regeneration. It was reported that PDLSC-derived CM (PDLSC-CM) promotes regenerative and anti-inflammatory effects in various tissues, including periodontal, chondrogenic, and osteogenic systems [2,3,7].

Because CM mirrors the state of the cell culture liquid, CM composition is affected by various physical and chemical preconditioning within the cell culture microenvironment [8]. Recent experiments have demonstrated that CM produced from cell cultures treated with certain bioactive molecules could modulate cellular activities in various experimental systems. For example, CM from macrophages incubated with a combination of several growth factors improved the phagocytosis of myelin debris and increased the expression of nerve repair-related genes in olfactory ensheathing cells [9]. CM derived from astrocytes cultured with ciliary neurotrophic factor was also found to contain potent neuroactive factors that significantly influence neuronal calcium channel activity [10]. In addition, CM from hypoxic effect-preconditioned human umbilical cord-derived mesenchymal stem cells was shown to induce increased secretion of angiogenic and neuro-protective factors relative to normal CM [11]. Although, to date, few reports have described the preconditioning processes for producing modified CM, particularly from PDLSCs, strategies for manufacturing PDLSC-CM with enhanced paracrine activities are required for the therapeutic use of cell-free CM in the field of periodontal regenerative medicine.

In several ways, modifying CM by adding specific chemical compounds to the culture medium can enhance specific cellular processes and target certain pathways to create desired therapeutic effects. Among the promising chemical candidates for CM production, this study explores chemicals, specifically in targeted periodontal tissue repair and regeneration. Metformin (1,1-dimethylbiguanide hydrochloride) is an oral anti-diabetic agent that is widely used for medication of type 2 diabetes [12]. It is further noted that this agent can promote the osteogenic differentiation of stem cells and osteogenic progenitor cells [12,13,14]. Regarding metformin’s effects on PDLSC activity, several studies recently demonstrated that direct treatment of PDLSC cultures with metformin promoted osteogenic differentiation [12,15,16]. Results from in vivo experimental models further showed accelerated formation of new bone in the peri-implant regions in animals treated with metformin compared with their control group, suggesting that metformin can enhance implant osseointegration [17,18]. When periodontitis was induced in a rat model, metformin treatment has shown attenuated inflammation and alveolar bone loss [19]. Emerging studies on diabetic periodontitis indicated that metformin encouraged reducing inflammation and less bone loss in periodontitis [20,21]. A clinical study also presented that applying gelated metformin into the periodontal pockets of patients with chronic periodontitis and bone defects significantly improved their clinical and radiological parameters [22].

Based on these previous works, metformin mainly exhibits dual capacities, with anti-diabetic coupled with anti-inflammation and osteogenic activities at both the cellular and tissue levels. Thus, it is worth challenging its considerable potential as a chemical preconditioning option to modify the culture conditions of PDLSCs, which could lead to a more targeted and potent secretome for promoting periodontal regeneration. However, not only has preconditioning PDLSCs with metformin rarely been reported, but it also has not been fully understood how metformin modifies the composition and functionality of the secreted factors and signaling molecules released from PDLSCs.

The present study hypothesized whether preconditioning PDLSC cultures with metformin could modify the content of CM toward increased levels of periodontal protective and regenerative factors and thus improve the therapeutic potential of normal PDLSC-CM for periodontal health. To test this hypothesis, this study was designed to (1) compare the secretory protein contents in modified CM obtained from metformin-preconditioned PDLSC culture (Met-CM) versus normal PDLSC-CM and (2) assess the effects of Met-CM on the differentiation capacity and molecular dynamics of PDLSCs under inflammatory conditions using RNA sequencing analysis. Our findings indicate that metformin preconditioning improved the secretome contents from PDLSCs and their CM product, and Met-CM promoted functional recovery in inflamed PDLSCs.

## 2. Materials and Methods

### 2.1. Periodontal Ligament Stem Cell Culture

Human PDLSCs (CELPROGEN, Torrance, CA, USA) were purchased and cultured in Minimum Essential Medium α (α-MEM; Gibco-BRL, Waltham, MA, USA) containing 10% fetal bovine serum (FBS; Gibco-BRL, Waltham, MA, USA) as previously reported [23]. The PDLSCs at passages 4–6 were used for all experiments. For each experiment, the basic α-MEM culture media was supplemented with 5% FBS, 50-μg/mL ascorbic acid, 1 μM dexamethasone, and 3 mM β-glycerophosphate, corresponding to the formula of osteogenic medium. Culture media were changed every 2 days. For experiments in which the PDLSC cultures were treated with Met-CM or lipopolysaccharide (LPS), cells were organized into the following four treatment groups: osteogenic medium (Control), osteogenic medium + LPS (5 μg/mL), Met-CM, and LPS + Met-CM. To correspond to the formula of osteogenic medium, Met-CM was complemented by the osteoinductive substances mentioned above for the Met-CM and LPS + Met-CM groups. All reagents and laboratory expendables were obtained from the Sigma Chemical Company (St. Louis, MO, USA) and SPL Lifescience (Pocheon, Republic of Korea), respectively.

### 2.2. Preparation of CM

PDLSC-CM and Met-CM were collected from cells grown with or without metformin (50 µM) under healthy culture conditions. In brief, PDLSCs were cultured to 70% confluency and then washed with phosphate-buffered saline (PBS), after which they were cultured with or without metformin in serum-free α-MEM in the absence of FBS and antibiotics for 48 h. Culture supernatants were then harvested and centrifuged at 3000 rpm for 5 min to remove cell debris. This obtained medium was referred to as PDLSC-CM or Met-CM for all experiments.

### 2.3. Protein Digestion

Prior to digestion, proteins were processed using filter-aided sample preparation on a Microcon 30 kDa Centrifugal Filter device (Millipore, Billerica, MA, USA) and reduced with Tris(2-carboxyethyl)phosphine at 37 °C for 30 min [24]. Proteins were alkylated with iodoacetic acid at 25 °C for 1 h in the dark and washed with lysis buffer and ammonium bicarbonate (50 mM). This sample was then treated and broken down into peptides with trypsin at 37 °C for 18 h. The peptides were desalted using C18 spin columns (Harvard Apparatus, Holliston, MA, USA) and eluted with 80% acetonitrile in 0.1% formic acid.

### 2.4. Liquid Chromatography–Tandem Mass Spectrometry (LC–MS/MS) Analysis

LC–MS/MS analysis was performed as described in a previous study [24]. Briefly, the peptide fragments were solubilized in 0.1% formic acid and analyzed using a Q-Exactive Orbitrap hybrid mass spectrometer (Thermo Fisher Scientific, Waltham, MA, USA) with an Ultimate 3000 system (Thermo Fisher Scientific). Peptides were separated according to their hydrophobicity using a 2 cm × 75 μm ID trap column packed with 3 μm C18 resin and a 50 cm × 75 μm ID analytical column packed with 2 μm C18 resin. After separation, data-dependent acquisition was performed, and the top 10 precursor peaks were selected for fragmentation. Ions were scanned at a high resolution (70,000 in MS1 and 17,500 in MS2 at *m/z* 400), with an MS scan range of 400–2000 *m/z* at both the MS1 and MS2 levels. Precursor ions were fragmented with 27% normalized collisional energy. Dynamic exclusion was adjusted to 30 s.

### 2.5. Proteome Data Analysis

Proteome data were analyzed as described in our previous study [25]. The MS/MS raw files of each analysis were explored using Proteome Discoverer™ software (ver. 2.5) and the Homo sapiens database (Uniprot). A peptide-spectrum match (PSM) identification and SEQUEST HT process were provided as a database search engine. The search parameters were established as follows: 10 ppm of tolerances of precursor ion masses, 0.02 Da fragment ion mass, and a maximum of two missed cleavages with trypsin. The dynamic modification of the peptide sequence was as follows: static carbamidomethylation of cysteine (+57.012 Da), variable modifications of methionine oxidation (+15.995 Da), acetylation of the protein’s N-term (+42.011 Da), and carbamylation of the protein’s N-term (+43.0006 Da). Among the accumulated findings, data below 1% of the FDR were selected and filtered at least 6 more peptide lengths.

### 2.6. Validation of DEPs by ELISA

An ELISA was performed as a validation tool for the differentially expressed proteins (DEPs) using an ELISA kit (AssayGenie, Dublin, Ireland). The experimental procedure was performed by following the manufacturer’s instructions. The optical density (OD) was measured spectrophotometrically at a wavelength of 450 nm.

### 2.7. Alkaline Phosphatase Activity

To assess the levels of PDLSC osteogenic differentiation, the alkaline phosphatase (ALP) activity was measured as previously described [23]. The extracted total was added to 200 μL of p-nitrophenyl phosphate (pNPP) and incubated for 30 min at 37 °C. The reaction was then stopped with 3-M NaOH solution, and the optical density was determined using spectrophotometry at 405 nm. ALP activity was presented in terms of mM/100 μg of protein.

### 2.8. Intracellular Calcium Quantification Assay

The intracellular calcium levels were measured as described in our previous report [23]. Briefly, cells were cultured in each experimental condition for 14 days, and the intracellular calcium concentration was measured using a QuantiChrom™ calcium assay kit (BioAssay Systems, Hayward, CA, USA) according to the manufacturer’s instructions. The calcium content of each sample was calculated in terms of mg/100 mg of protein, from which the optical density (OD) was measured at 612 nm.

### 2.9. RNA Extraction and Real-Time Reverse Transcription (qRT)-PCR

To examine the mRNA expression of osteogenic and cementogenic factors, and to validate the RNA sequencing results, RNA extraction, cDNA synthesis, and qRT-PCR were performed as previously described [23] and according to the instructions provided with the QuantiTect SYBR Green PCR Kit (QIAGEN) using an iCycler iQ Multi-Color Real-Time Detection System (Bio-Rad). The thermal cycling conditions were set to 95 °C for 30 s, 95 °C for 5 s, 55 °C for 30 s, and 72 °C for 30 s for 30 cycles. The primers were as follows: 5′-CCCAGTATGAGAGTAGGTGTCC-3′ (sense) and 5′-GGGTAAGACTGGTCATAGGACC-3′ (antisense) for *RUNX2*; 5′-CGCTACCTGTATCAATGGCTGG-3′ (sense) and 5′-CTCCTGAAAGCCGATGTGGTCA-3′ (antisense) for *OCN*; 5′-TTCTGCGGCAAGAGGTTCACTC-3′ (sense) and 5′-GTGTTTGCTCAGGTGGTCGCTT-3′ (antisense) for *OSX*; 5′-CCATCCTATCTCTTTGGACCTGG-3′ (sense) and 5′-CCTTGCTTACAGGTGCTGTCCT-3′ (antisense) for *CEMP-1*; and 5′-GCTCTCCAGAACATCATCC-3′ (sense) and 5′-TGCTTCACCACCTTCTTG-3′ (antisense) for *GAPDH*; 5′-ATGGTCACCTGGTCACTCCAAC-3′ (sense) and 5′-GAGGCACAGAAGCTGCAAAAGG-3′ (antisense) for *OPN3*; 5′-TGTATCTGCTCTCGGACAAGGC-3′ (sense) and 5′-GCTGAGGAAACTGCATTGGAACC-3′ (antisense) for *TRPM4*; 5′-CACTACCATCTGAACTGTGGCTG-3′ (sense) and 5′-GCTTTCGTTCCAACAGCCAGTC-3′ (antisense) for *TIMP4*; and 5′-GGCACAATGTCTCCTCCAGAGA-3′ (sense) and 5′-CAGATGAAGCCTTGGTCAGTGC-3′ (antisense) for *FOXP3*.

### 2.10. Library Preparation and Sequencing

The total RNA was extracted, and the RNA concentration was assessed. The construction of a library was prepared using a QuantSeq 3′ mRNA-Seq Library Prep Kit FWD (Lexogen) according to the manufacturer’s instructions [26]. In brief, reverse transcription was conducted with the hybridized product of the prepared total RNA of each sample and an oligo-dT primer with an Illumina-compatible sequence at its 5′ end. The RNA template was then degraded, and the second strand was initiated to synthesize with a random primer containing an Illumina-compatible linker sequence at its 5′ end. The double-stranded library was purified and amplified to add the necessary adapter sequences for cluster generation. The completed library was then purified to remove contamination from the PCR components. Eventually, high-throughput sequencing was performed in the form of single-end 75 bp sequencing using NextSeq 500 (Illumina Inc., San Diego, CA, USA).

### 2.11. Genome Data Analysis

FastQC was used to evaluate the quality control of the raw sequencing data [27]. Adapter sequences and low-quality reads were removed by using bbduk 38.34 [28]. The clean sequencing reads were aligned to a known reference genome sequence using Bowtie2 alignment software, specifically version 2.3.5.1 [29]. The reads were then quantified using Bedtools v2.27.1 [30]. The TMM+CPM normalization method using EdgeR was applied to the read counts [31]. Data mining and graphic visualization were established using ExDEGA (Ebiogen Inc., Seoul, Republic of Korea). The DAVID (http://david.abcc.ncifcrf.gov/, accessed on 31 January 2023) program was used to determine the gene classification, ontology, and Kyoto Encyclopedia of Genes and Genomes (KEGG) pathways. STRING v3.8.2 was employed to predict protein–protein interactions (PPIs), with a PPI score >0.4 (*p* < 0.05) considered to be significant. The PPI networks were also visualized using Cytoscape v3.10.0 software (http://www.cytoscape.org, accessed on 2 November 2023).

### 2.12. Statistical Analysis

Data are shown as the mean ± SD (n ≥ 3), and they were evaluated using the Student’s *t*-test. One-way analysis of variance was performed for multiple comparisons using Duncan’s multiple range test, where *p* values < 0.05 were considered statistically significant. The figures shown are representative of the data.

## 3. Results

### 3.1. Comparison of Differentially Expressed Proteins (DEPs) in PDLSC-CM Versus Met-CM

**Figure 1:**
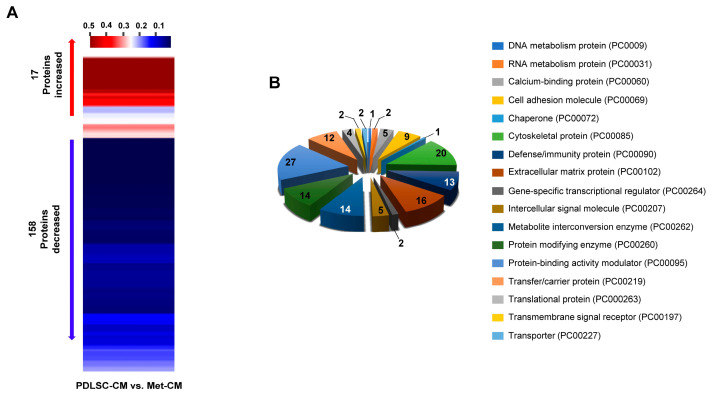
Comparative proteomic analysis of proteins in condition medium (CM) from periodontal ligament stem cells (PDLSCs; PDLSC-CM) vs. those in CM from metformin-treated PDLSCs (Met-CM). (**A**) Hierarchical clustering of significantly differentially expressed proteins (fold-change ≥2 and *p* < 0.05). Colors depict relative expression levels of proteins, with red and blue indicating up- and downregulation, respectively. (**B**) Classification analysis of candidate proteins, categorized using the PANTHER program. The *x* axis shows the protein classification illustrated by different colors, and the number of proteins within each category is plotted on the *y* axis.

**Figure 2:**
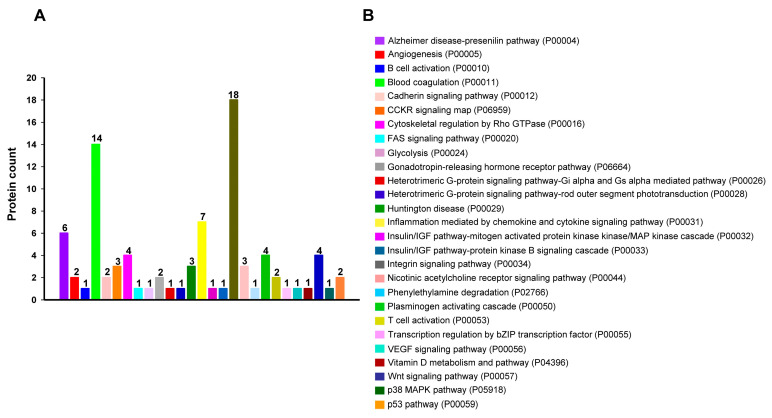
Signaling pathway enrichment analysis for 88 of the 175 differentially expressed proteins (DEPs) using the PANTHER program. (**A**) Distribution of proteins and (**B**) the list of pathways identified by PANTHER.

To identify proteins differentially expressed in Met-CM compared with PDLSC-CM, we collected CM from cultures of human PDLSCs grown in normal or metformin-preconditioned serum-free culture media. LC–MS/MS proteomics was then performed, uncovering a total of 202 DEPs. We further analyzed the DEPs and identified 175 significant protein changes based on a fold-change cut-off of ≥2 and *p* < 0.05 (Supplementary Data S1). Hierarchical clustering was performed to group significant DEPs, and dynamic changes in the DEPs were assessed, revealing that 17 proteins were increased and 158 proteins were decreased in Met-CM relative to PDLSC-CM (Figure 1A). The enzyme-linked immunosorbent assay (ELISA) results were used to validate the DEPs. The ELISA results yielded consistency with the proteomics data for three proteins: fibronectin, keratin type II cytoskeletal 1 (KRT1), and caspase-14 (Supplementary Data S2A). Subsequently, we used PANTHER to predict the potential roles of these proteins, and 149 of the 175 candidate DEPs recognized by the software were categorized based on their known functions (Figure 1B). Among the analyzed groups, the top three protein classes in 17 categories were the protein-binding activity modulator (PC00095), cytoskeletal protein (PC00085), and extracellular matrix (ECM) protein (PC00102). These protein groups were also identified by PANTHER subgroup classification (Supplementary Data S3). In total, 88 of the 175 DEPs recognized by PANTHER were assigned to molecular pathways, including integrin signaling, cytoskeletal regulation by Rho GTPase, and the Wnt signaling pathway (Figure 2). These comparative proteomics results indicate that the cellular secretome in Met-CM comprised many categories of proteins with the potential to modulate the molecular function of PDLSCs.

### 3.2. Effect of Met-CM on the Differentiation Capability of PDLSCs in LPS-Induced Inflammatory Conditions

**Figure 3:**
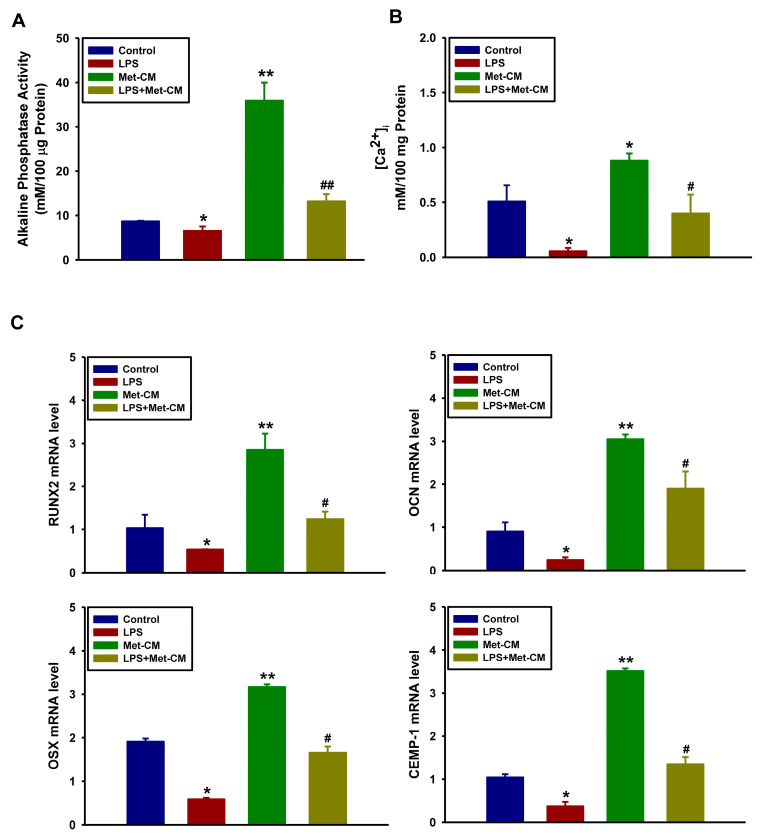
Effects of lipopolysaccharide (LPS) and metformin-preconditioned periodontal ligament stem cell (PDLSC) medium (Met-CM) on the osteogenic differentiation of PDLSCs. (**A**) Alkaline phosphatase (ALP) activity was measured after cells were cultured with LPS, Met-CM, or LPS + Met-CM for 7 days, and (**B**) [Ca^2+^]_i_ was also assessed in same culture conditions for 14 days, as described under Materials and Methods. (**C**) The mRNA expression levels of *RUNX2*, *OCN*, *OSX*, and *CEMP-1* were assessed by real-time reverse transcription (qRT)-PCR after 7 days of osteogenic induction. The values are shown as the mean ± standard deviation (n = 5). * *p* < 0.05 and ** *p* < 0.001 vs. the control value. ^#^ *p* < 0.05 and ^##^ *p* < 0.001 vs. the Met-CM value at each time point.

We next evaluated the effect of Met-CM on the osteogenic and cementogenic differentiation of PDLSCs in an in vitro inflamed microenvironment. To this end, the cells were treated with osteogenic medium (as a control), osteogenic medium + LPS, Met-CM, or LPS + Met-CM. We then measured the osteogenic differentiation and found it to be significantly decreased in cells treated with LPS relative to the control group, as determined by ALP activity (Figure 3A) and intracellular calcium levels ([Ca^2+^]_i_) (Figure 3B). In addition, the mRNA expression levels of osteogenic and cementogenic factors, such as *RUNX2*, *OCN*, *OSX*, and *CEMP-1* (Figure 3C), were downregulated by LPS treatment. In contrast, treatment with Met-CM significantly increased the osteogenic and cementogenic activity of PDLSCs compared with the control group. In addition, treatment with Met-CM alleviated the downregulation of osteogenic and cementogenic differentiation observed in the inflamed PDLSCs treated with LPS. These results indicate that Met-CM contains a PDLSC secretome that can efficiently heighten the differentiation potential of PDLSCs and therefore potentially influence periodontal tissue repair and regeneration.

### 3.3. Identification of DEGs and Gene Ontology (GO) Enrichment

**Figure 4:**
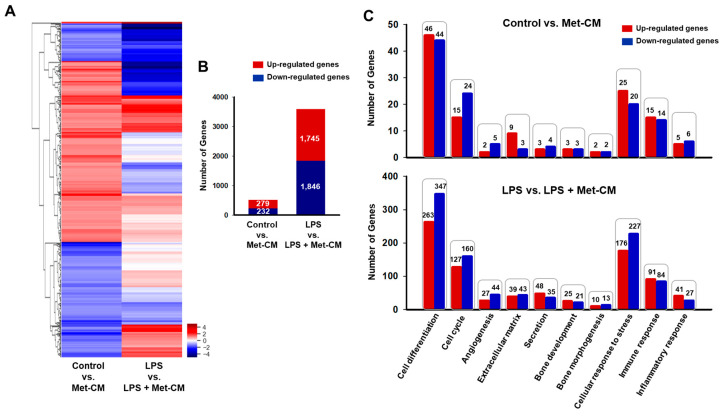
RNA sequencing of periodontal ligament stem cells (PDLSCs) cultured with osteogenic medium, osteogenic medium + lipopolysaccharide (LPS), metformin-preconditioned PDLSC medium (Met-CM), or LPS + Met-CM. (**A**) Heat map of significantly differentially expressed genes (DEGs) (fold-change ≥2 and *p* < 0.05). (**B**) Numbers of up- and downregulated DEGs in PDLSCs. Red indicates upregulation, and blue indicates downregulation. (**C**) Functional gene categories in each comparison.

**Figure 5:**
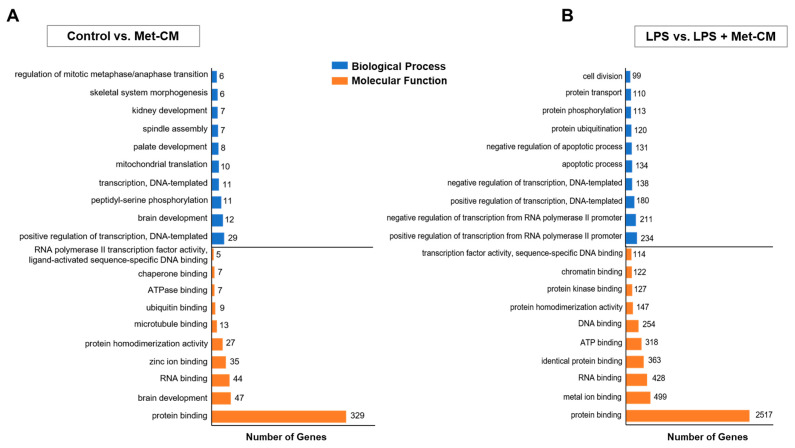
Top 10 Gene Ontology (GO) terms from the biological processes and molecular functions enriched among differentially expressed genes (DEGs) identified from the (**A**) control vs. metformin-preconditioned periodontal ligament stem cell (PDLSC) medium (Met-CM) and (**B**) lipopolysaccharide (LPS) vs. LPS + Met-CM comparisons using GO analysis in DAVID (*p* < 0.05).

To further investigate the effect of Met-CM on the global expression of genes and molecular pathways in PDLSCs, the cells in the control, LPS-, Met-CM-, and LPS + Met-CM-treated groups were analyzed by RNA sequencing. We then performed comparative analyses of the DEGs (fold-change ≥2 and *p* < 0.05) in the control versus Met-CM and LPS versus LPS + Met-CM groups. From these two comparisons, we identified 511 (279 upregulated and 232 downregulated) and 3591 (1745 upregulated and 1846 downregulated) DEGs, respectively (Figure 4A,B, Supplementary Data S4). The genomic profile was then validated through qRT-PCR, confirming the results obtained from RNA sequencing (Supplementary Data S2B). We then classified the DEGs into specific functional categories, including cell differentiation, cell cycle, angiogenesis, ECM, secretion, bone development, bone morphogenesis, cellular response to stress, immune response, and inflammatory response in the different comparisons (Figure 4C). The DEGs identified from each comparison were also analyzed by GO classification to identify enriched biological processes and molecular functions (Figure 5, Supplementary Data S5 and S6). The DEGs were specifically enriched in biological processes, including positive regulation of DNA-templated transcription and skeletal system morphogenesis in the control versus Met-CM comparison and positive regulation of transcription from the RNA polymerase II promoter and negative regulation of the apoptotic process in the LPS versus LPS + Met-CM comparison. Molecular function analysis revealed the enrichment of protein-binding terms among the DEGs from each comparison. These comparative analyses of the DEGs identified above, combined with functional annotation, reveal the molecular biomarker profiles produced in response to Met-CM treatment in healthy or inflamed PDLSC cultures.

### 3.4. KEGG Enrichment Pathway Analysis

The signaling pathways enriched among the DEGs identified from the two comparisons were further analyzed using the DAVID program; the top 10 significantly enriched pathways from each comparison are shown in Table 1.

**Figure 6:**
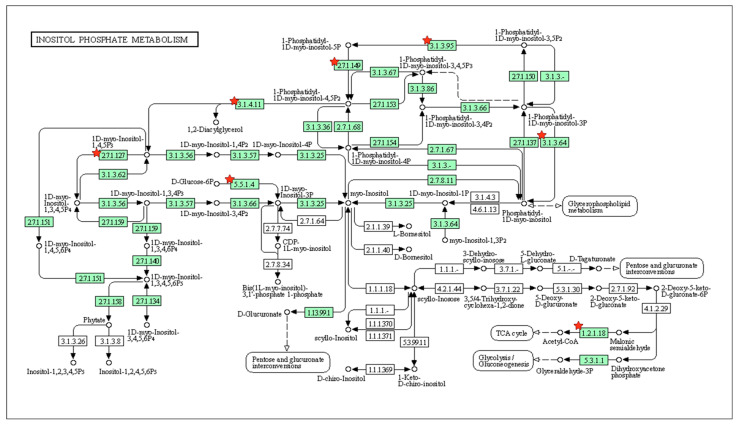
Image of the inositol phosphate metabolism pathway enriched among the imported DEGs identified from the control vs. metformin-preconditioned periodontal ligament stem cell (PDLSC) medium (Met-CM) comparison. The red stars indicate key genes.

**Figure 7:**
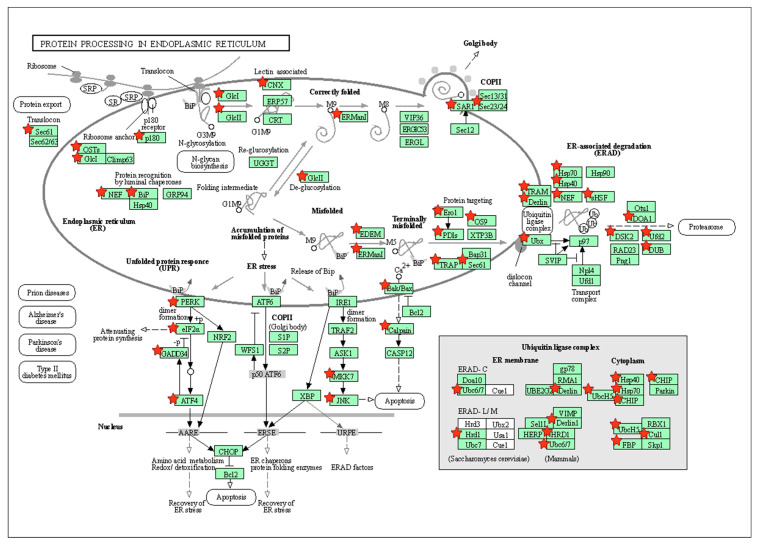
Images of the protein processing in the endoplasmic reticulum pathways enriched among the imported differentially expressed genes (DEGs) identified from the lipopolysaccharide (LPS) vs. LPS + Met-CM comparison. The red stars indicate key genes.

Among the KEGG pathways identified, intriguing candidates with a possible role in bone and tooth mineralization and repair [32,33] included inositol phosphate metabolism for DEGs from the control versus Met-CM comparison (Figure 6) and protein processing in the endoplasmic reticulum (ER) for DEGs from the LPS versus LPS + Met-CM comparison (Figure 7).

### 3.5. Identification of PPI Networks

Lastly, we uploaded the DEGs from the two comparisons to the STRING online database and constructed putative PPI networks for each group. We detected 491 nodes (representing proteins) and 857 edges (representing interactions) in the control versus Met-CM network and 3427 nodes and 58,791 edges in the LPS versus LPS + Met-CM network. The top five hub proteins in each comparison are listed in Table 2.

**Figure 8:**
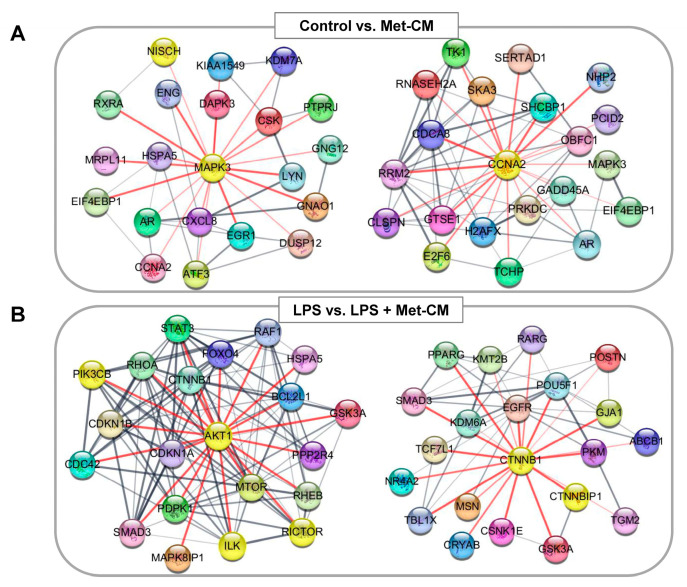
Protein–protein interaction (PPI) network analysis using the STRING database. (**A**) The MAPK3 and CCNA2 hub networks for differentially expressed genes (DEGs) identified from the control vs. metformin-preconditioned medium (Met-CM) comparison. (**B**) The AKT1 and CTNNB1 hub networks for DEGs identified from the lipopolysaccharide (LPS) vs. LPS + Met-CM comparison. Each node marks a protein, and each edge designates an interaction. A confidence (interaction) score of at least 0.4 was set for significance. The PPI network was organized and visualized using Cytoscape v3.10.0.

From these PPI network data, we identified and visualized the meaningful clusters in each comparison, including mitogen-activated protein kinase 3 (MAPK3) and cyclin A2 (CCNA2) in the control versus Met-CM group and AKT1 substrate 1 (AKT1) and catenin beta 1 (CTNNB1) in the LPS versus LPS + Met-CM group using Cytoscape software (Figure 8).

## 4. Discussion

This study has shown how metformin modifies and potentiates the secretome and CM of PDLSCs and Met-CM encourages functional recovery of inflamed PDLSCs. Regarding the potent secretome contents to advance periodontal regeneration, comparative proteomics analysis revealed a high proportion of cytoskeletal and ECM proteins in Met-CM compared with the normal PDLSC-CM. These findings may be related to the signaling pathways identified, including integrin signaling, cytoskeletal regulation by Rho GTPase, and the Wnt signaling pathway. It is well known that the cytoskeleton, including microtubules, actin fibers, and intermediate filaments, interacts with proteins of the ECM in cell-to-matrix and cell-to-cell adhesions, as well as in various cellular processes, such as proliferation, migration, differentiation, and apoptosis [34]. In periodontal tissues, resident stem and progenitor cells commonly induce complex signaling pathways that regulate cellular components ranging from the ECM to the cytoskeleton and the nucleus, regarded as mechanoresponsive cellular processes [35,36,37]. Furthermore, the interplay among periodontal ECM components and cytoskeletal structures contributes to periodontal health and tissue regeneration [38,39]. Thus, our results suggest that the Met-CM containing concentrated cytoskeletal and ECM contents could serve as a possible therapeutic agent for periodontal tissue diseases and oral regenerative medicine.

This study further demonstrated the ability of Met-CM to promote the recovery of activities in inflamed PDLSCs. Using genomic profiling, we also analyzed the biological processes and dynamic molecular pathways enriched among DEGs identified from two comparative groups—PDLSCs treated with the control versus Met-CM and LPS versus LPS + Met-CM—to clearly define the effects of Met-CM in healthy or inflamed PDLSCs. KEGG pathway analysis revealed that the DEGs in the PDLSCs treated with Met-CM versus the control group were enriched for inositol phosphate metabolism. The inositol-derived metabolites, known as inositol polyphosphates, have a broad range of physiological functions and cellular activities, including DNA damage repair, energy production, and calcium homeostasis [40,41,42]. Numerous prior studies have suggested that metformin acts through the inositol phosphate signaling network and the phospholipase C/inositol 1,4,5-trisphosphate (IP3)/Ca^2+^ signaling pathway [43,44], which is also known to be a critical cellular cascade for bone and tooth mineralization and formation [45,46].

Our KEGG analysis also identified the enrichment of protein processing in the ER in LPS-stimulated PDLSCs treated with Met-CM. Protein processing in the ER ensures correct protein conformation and quality for maintaining protein homeostasis [47]. Protein homeostasis can be disrupted in cells responding to a variety of pathological and physiological conditions that induce ER stress. Consequently, cells will attempt to restore homeostasis through the downstream unfolded protein response (UPR) pathway, which comprises protein kinase-R-like ER kinase (PERK), inositol-requiring enzyme 1α (IRE1α), and activating transcription factor 6 (ATF6) [48]. Previous studies have shown that the IRE1α-X-box binding protein (XBP1), PERK-eukaryotic initiation factor 2α (eIF2α), and ATF6 signaling pathways induce the UPR and restore ER stability [49,50]. However, the role of ER stress signaling pathways in the periodontal healing process is not clearly defined. In contrast, most studies have reported that ER stress signaling pathways are involved in cell apoptosis and death and act as key players in various human diseases [51,52,53]. One recent study reported that ER stress stimulates osteogenic responses and alveolar bone formation in tooth extraction sockets [33]. Moreover, another interesting investigation found that ER stress-activated PERK-eIF2α-ATF4 pathways promote osteogenic differentiation of human periodontal ligament cells under conditions of mechanical stress [46]. Our results here, together with those from previous studies, may indicate that the ER system is dynamically regulated and influenced in unconventional ways under specific pathological conditions, resulting in the healing or degeneration of periodontal tissue. However, further research is required to elucidate the possible association between the ER organelle and periodontal biological signaling.

The results from our PPI network analysis further identified MAPK3 and cyclin A2 in the control versus Met-CM-treated PDLSCs. These highly interacting and abundant proteins are widely known as signaling molecules that regulate various cellular processes, such as proliferation, differentiation, and cell cycle progression, in response to various extracellular signals [54,55,56]. In the LPS versus LPS + Met-CM groups, the notable hub proteins identified were AKT1 and CTNNB1, which are regarded as positive regulators of osteogenic differentiation of stem cells and bone mineralization and formation, as well as embryonic development and organogenesis and adult tissue homeostasis [57,58,59,60]. These clustering analyses could indicate that Met-CM treatment in healthy or inflamed PDLSC cultures promotes enhanced tissue growth and regeneration dynamics, suggesting potential therapeutic roles and biomarkers for periodontal diseases and defects.

Although this study suggests novel Met-CM efficacy in periodontal regeneration, it is still limited within the scope of an in vitro periodontal model. Given that periodontal biological and pathological events are regulated by the complex interactions between cells and their resident tissue environments, further research would be required to validate the impact of Met-CM on periodontal tissues with preclinical or clinical periodontal disease. While these proteomics and genomics studies confirmed various proteins, genes, and signaling pathways which could be involved in restoring impaired functions of PDLSCs, further examination with the interconnecting mechanisms between individual molecules could strengthen the present findings. To obtain more abundant protein or gene outcomes from the proteomics and genomics techniques, a valid strategy to produce concentrated formulations of CM can be employed in further experiments.

In conclusion, these findings suggest that Met-CM could represent a beneficial formula with regenerative potential for periodontal tissue and highlight encouraging functional recovery of inflamed PDLSCs. The modified and enriched PDLSC-CM as a cell-free product can be used in various periodontal therapeutic applications, including tissue engineering, cell-based therapies, and regenerative dentistry after the establishment of quality control criteria and manufacturing pharmaceutical standardization for the intended composition and concentration of CM.

## Figures and Tables

**Table 1:** KEGG pathway enrichment analysis.

	ID	Term	Count Gene	*p* Value
Control vs. Met-CM				
	hsa05225	Hepatocellular carcinoma	12	7.82 × 10^−4^
	hsa05142	Chagas disease	9	0.001
	hsa05200	Pathways in cancer	22	0.004
	hsa05226	Gastric cancer	10	0.004
	hsa00562	Inositol phosphate metabolism	7	0.004
	hsa00600	Sphingolipid metabolism	6	0.005
	hsa05216	Thyroid cancer	5	0.007
	hsa05217	Basal cell carcinoma	6	0.010
	hsa05221	Acute myeloid leukemia	6	0.013
	hsa05417	Lipid and atherosclerosis	11	0.015
LPS vs. LPS + Met-CM				
	hsa04932	Non-alcoholic fatty liver disease	61	2.45 × 10^−10^
	hsa04141	Protein processing in endoplasmic reticulum	65	3.25 × 10^−10^
	hsa05012	Parkinson disease	88	7.75 × 10^−10^
	hsa05014	Amyotrophic lateral sclerosis	111	8.69 × 10^−10^
	hsa04144	Endocytosis	83	2.57 × 10^−9^
	hsa05208	Chemical carcinogenesis—reactive oxygen species	76	2.96 × 10^−9^
	hsa05020	Prion disease	88	3.29 × 10^−9^
	hsa05016	Huntington disease	95	6.23 × 10^−9^
	hsa05022	Pathways of neurodegeneration—multiple diseases	132	1.58 × 10^−8^
	hsa05210	Colorectal cancer	38	2.67 × 10^−8^

**Table 2:** Top hub proteins with the highest number of PPIs.

Gene Name	Protein Description	No. of Interacting Proteins
Control vs. Met-CM		
MAPK3	mitogen-activated protein kinase 3	23
CCNA2	cyclin A2	20
MRPL4	mitochondrial ribosomal protein L4	20
UTP18	UTP18, small subunit processome component	16
NIP7	NIP7, nucleolar pre-rRNA processing protein	16
LPS vs. LPS + Met-CM		
ACTB	actin, beta	464
GAPDH	glyceraldehyde-3-phosphate dehydrogenase	413
AKT1	AKT1 substrate 1	409
CTNNB1	catenin beta 1	352
UBA52	ubiquitin A-52 residue ribosomal protein fusion product 1	345

## Data Availability

The original contributions presented in this study are included in the article and Supplementary Materials. Further inquiries can be directed to the corresponding author.

## Supplementary Materials

The following supporting information can be downloaded at: https://www.mdpi.com/article/10.3390/jfb16050177/s1, Supplementary Data S1: LC-MS/MS data; Supplementary Data S2A: ELISA results to validate the DEPs; Supplementary Data S2B: qRT-PCR data to validate the DEGs; Supplementary Data S3: PANTHER subgroup classification; Supplementary Data S4; RNA sequencing-DEG data; Supplementary Data S5: GO classification to identify enriched biological processes; Supplementary Data S6: GO classification to identify enriched molecular functions.
