# Supplementary figures and images for: Metformin-Enhanced Secretome from Periodontal Ligament Stem Cells Promotes Functional Recovery in an Inflamed Periodontal Model: In Vitro Study
Source: J Funct Biomater. 2025 May 13;16(5):177. doi: 10.3390/jfb16050177 (PMC12112599; doi:10.3390/jfb16050177)

## Slide 1
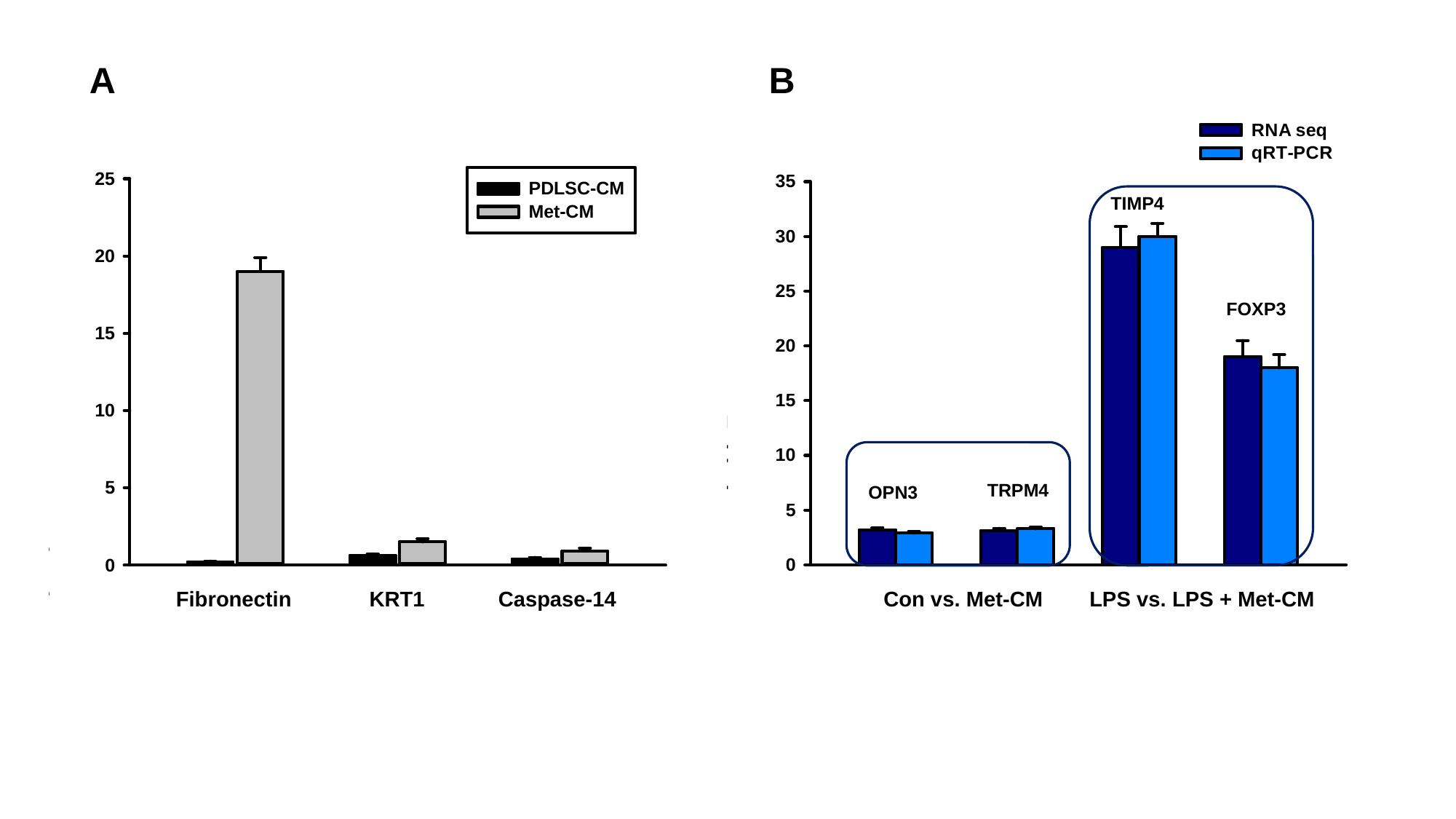

A
B
TIMP4
FOXP3
TRPM4
OPN3
Fibronectin
KRT1
Caspase-14
Con vs. Met-CM
LPS vs. LPS + Met-CM

Supplement: Supplementary file 1 [file jfb-16-00177-s001.zip › Supplementary data S2.pptx]
